# A method to disentangle and quantify host anabolic turnover in photosymbiotic holobionts with subcellular resolution

**DOI:** 10.1038/s42003-019-0742-6

**Published:** 2020-01-08

**Authors:** Emma Gibbin, Guilhem Banc-Prandi, Maoz Fine, Arnaud Comment, Anders Meibom

**Affiliations:** 1 Laboratory for Biological Geochemistry, School of Architecture, Civil and Environmental Engineering, École Polytechnique Fédérale de Lausanne, Lausanne, Switzerland; 2 The Interuniversity Institute for Marine Sciences, 88103 Eilat, Israel; 3 The Mina and Everard Goodman Faculty of Life Sciences, Bar-Ilan University, 52900 Ramat-Gan, Israel; 4 Cancer Research UK Cambridge Institute, University of Cambridge, Robinson Way, Cambridge, CB2 0RE UK; 5 Center for Advanced Surface Analysis, Institute of Earth Sciences, University of Lausanne, Lausanne, Switzerland

**Keywords:** Biological techniques, Microbiology, Mass spectrometry

## Abstract

A wide range of organisms host photosynthesizing symbionts. In these animals the metabolic exchange between host and symbionts has prevented in situ host anabolic turnover to be studied without the confounding effect of translocated photosynthates. Using the symbiotic coral *Stylophora pistillata* as a model organism and [1-^13^C]-pyruvate and [2,3-^13^C]-pyruvate in different incubation conditions (light, light + DCMU, and darkness), we employed NanoSIMS isotopic imaging to quantify host anabolism, with and without translocated metabolites from their photosynthesizing dinoflagellate symbionts. Under our experimental conditions, host de novo lipid synthesis accounted for ~40% of the total holobiont lipid reserve, and dinoflagellate recycling of metabolic ^13^CO_2_ enhanced host tissue ^13^C-enrichment by 13–22% in the epidermis, 40–58% in the gastrodermis, and 135–169% in host lipid bodies. Furthermore, we show that host anabolic turnover in different tissue structures differs, in a manner consistent with the localisation, function and cellular composition of these structures.

## Abstract (web summary)

Gibbin et al. use [1–13C]-pyruvate and [2,3–13C]-pyruvate in different incubation conditions (light, light+DCMU, and dark) to discern and quantify coral host anabolism, with and without translocated metabolites from their photosynthesizing symbionts.

## Introduction

Aerobic cell metabolism consists of two complementary processes: catabolism, i.e., the ATP-producing oxidation of complex carbon molecules into CO_2_, and anabolism, i.e., the synthesis of building block molecules into compounds that have higher structural complexity. The tricarboxylic acid (TCA) cycle in mitochondria drives both catabolic and anabolic reactions^1^. Pyruvate is the primary metabolite feeding the TCA cycle. The pyruvate molecule contains three carbon (C) atoms, and the fate of each of these C atoms differs. Upon entering the TCA cycle, the C-atom in position 1 is lost as CO_2_ during the formation of Acetyl-CoA, which subsequently delivers the other two pyruvate-derived C atoms to the TCA cycle. Once in the TCA cycle, the fate of the two remaining C atoms depends on the energetic state of two enzymes: isocitrate dehydrogenase and α-ketoglutarate dehydrogenase. If demand for energy is high, the two C-atoms are oxidized to form CO_2_ in the process that generates nicotinamide adenine dinucleotide (NADH) and drives ATP synthesis via oxidative phosphorylation. Conversely, if energy demand is low, molecular synthesis is favoured, and the two C-atoms are incorporated into intermediate molecules, which act as the pre-cursers of carbohydrates, proteins, and lipids^1,2^. It is this latter transfer of pyruvate-derived C into the anabolic molecular products of the TCA cycle that forms the basis for the method presented here.

Recent advances in ion microprobe (NanoSIMS) imaging allows the in situ distribution of enriched stable isotopes in biological tissue to be imaged at a spatial resolution of ~100 nm. The resulting isotopic maps can be correlated directly with ultrastructural observations of the cell by electron microscopy (EM) of the same thin or semi-thin section^3–5^. The classical sample preparation requirements for EM and NanoSIMS involve a number of steps that remove the soluble parts of the cell (i.e., the cytosol). However, the structural components of the cell (i.e., proteins, fatty acids, RNA, DNA, etc.), which represent the products of anabolic metabolism and to a large extent originate from the TCA cycle, remain in place. Combined with isotopic labelling of precursor molecules, the isotopic enrichments quantified through NanoSIMS imaging therefore show the formation of new structural components, i.e., the anabolic turnover. Comparing samples that have received the same amount of isotopically labelled substrate permits the comparison of anabolic activity between individual cells and/or tissues.

Corals are meta-organisms (so-called holobionts^6^) that host a dynamic population of bacteria, archaea, viruses and photosynthetic dinoflagellate algae (family: Symbiodiniaceae)^7^. The flux of metabolites between the host and its symbionts has been the subject of numerous studies using stable isotope labelling combined with either bulk tissue analyses^8,9^ or correlative imaging^10–15^. However, these previous studies have generally focused on the photosynthetic performance of the algae and the metabolic interactions between host and symbiont population. To date, it has not been possible to investigate the anabolic performance of the coral host tissue without the translocation of photosynthates from the symbiont algae population. Here we demonstrate this capability using pyruvate-labelled with ^13^C in specific C-positions within the molecule. This method is not only applicable to corals, but can be used to study a wide range of organisms that host photosynthesizing symbionts: e.g., bivalves, gastropods, jellyfish, sea anemones, hydrozoans, soft corals, foraminifera, sponges, and worms.

## Results and discussion

In organisms hosting photosynthesizing symbionts, both the ^13^CO_2_ produced as a by-product of the formation of the Acetyl-CoA complex (C-position 1) and during the TCA cycle (C-positions 2 and 3) can be assimilated by the symbionts. Photosynthates that are labelled in ^13^C and translocated back to the host tissue then make it impossible to obtain a measure of pure host-cell anabolic turnover. This problem of photosynthate translocation can be avoided either by conducting experiments in the dark and/or by blocking photosynthesis, rendering it impossible for translocated photosynthates to contribute to ^13^C-enrichment in the host tissue. Here we have used symbiotic, reef-building corals as the model system to illustrate these strategies.

### Labelling patterns produced by [1-^13^C]-pyruvate

**Fig. 1:**
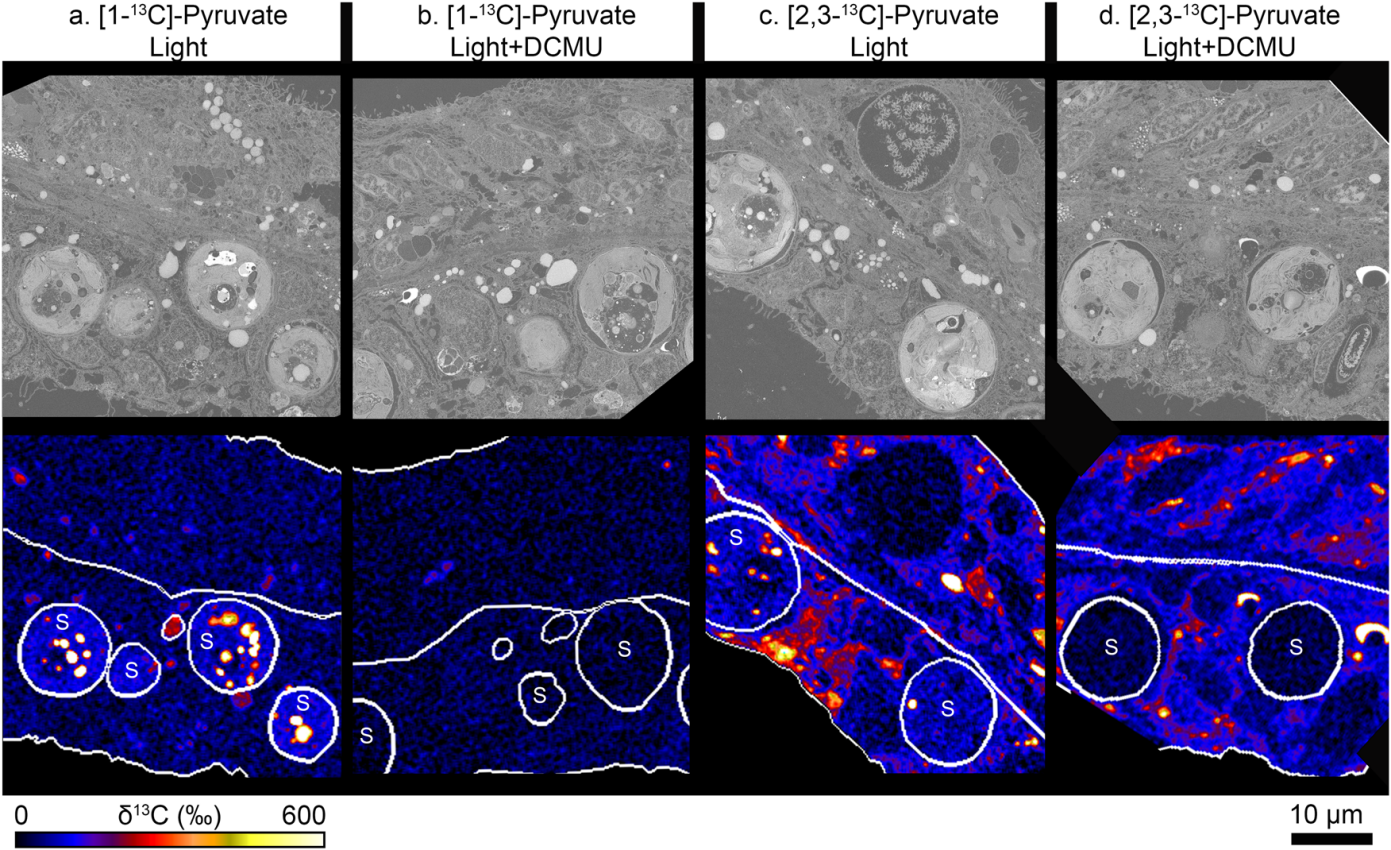
Isolating host anabolism using position-specific ^13^C-labelling of pyruvate. Corals were incubated with differentially labelled pyruvate, in the presence or absence of the photosynthetic inhibitor DCMU, for 12 h. Scanning electron microscopy (top row) images and their correlative NanoSIMS image (bottom row) are shown for corals labelled with **a** [1-^13^C]-pyruvate in the light; **b** [1-^13^C]-pyruvate in the light + DCMU; **c** [2,3-^13^C]-pyruvate in the light, and **d** [2,3-^13^C]-pyruvate in the light + DCMU. Circles labelled with ‘S’ show the position of the algal symbionts in their hosts’ oral gastrodermis, while unmarked circles show the position of host lipid bodies. Note that only a couple of host lipid bodies are circled for illustration purposes; these ROIs do not reflect the real abundance of lipids in the tissue, all of which were included in the analysis. See text for an explanation of the differential labelling patterns.

**Fig. 2:**
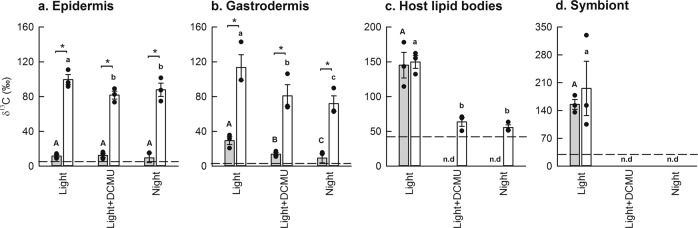
Quantitative analysis of anabolism following incubation with position-specific ^13^C-labelled pyruvate. Shown is the relative assimilation of ^13^C (mean ± SE of *n* = 3 coral colonies) after exposure to either [1-^13^C]-pyruvate (grey) or [2,3-^13^C]-pyruvate (white), in the host epidermis **a**; host gastrodermis **b**; host lipid bodies **c**; and algal symbionts **d**. Incubations were conducted under several treatments: in the light, with and without the inhibition of photosynthesis (i.e., ±DCMU), and at night. Asterisks denote significant differences (*α* = 0.004) between pyruvate types, within a treatment. Majuscule letters show significant differences between treatments in corals exposed to [1-^13^C]-pyruvate, and lowercase letters show significant differences between treatments in corals exposed to [2,3-^13^C]-pyruvate. The dashed line represents the sensitivity threshold of the NanoSIMS for each region of interest (calculated as the mean + 3-sigma, of the same area in an identically prepared unlabelled control). If ^13^C-‘enrichments’ fell below this limit it was considered not detectable (n.d.).

When photosynthesis was active, [1-^13^C]-pyruvate produced ^13^C-labelling patterns qualitatively similar to those previously observed in corals exposed to ^13^C-labelled bicarbonate in seawater, with preferential accumulation in the pyrenoid and starch granules of the dinoflagellates, and translocated ^13^C-labelled lipids in the coral host tissue^10,13,15^ (Fig. 1a). As expected, when photosynthesis was inactive, i.e., in the light + DCMU and night treatments, no ^13^C-assimilation was detected in either the symbionts or the host lipid bodies (Figs. 1b and  2c, d). However, weak and diffuse ^13^C-labelling was observed in both the oral epidermis and gastrodermis in all incubation conditions (Fig. 2a, b). This may result from [1-^13^C]-pyruvate redirected through gluconeogenic pathways, but this background-labelling level is insignificant compared to those obtained with [2,3-^13^C]-pyruvate under similar conditions.

### Labelling patterns produced by [2,3-^13^C]-pyruvate

Incubation with [2,3-^13^C]-pyruvate resulted in strong, heterogeneously distributed ^13^C-labelling of the host tissues in all conditions (Figs. 1c, d and  2a, b). The similarity of ^13^C-assimilation levels in the epidermis (seawater-facing) and gastrodermis (coelenteron-facing) in the light + DCMU and night treatments supports the assumption that tissue layers have access to, and assimilate pyruvate equally (Supplementary Fig. 1), consistent with the recent observation of ubiquitous (pyruvate-transporting) monocarboxylate transporters in cnidarians^16^. The total absence of ^13^C-labelling in the symbionts in the light + DCMU incubation (Figs. 1d and 2d) was expected, because dinoflagellate algae lack the pyruvate dehydrogenase complex required to breakdown pyruvate into acetyl-CoA^17^. They are thus unable to use pyruvate directly to fuel their own TCA cycle, or produce lipids.

The [2,3-^13^C]-pyruvate incubations permit several novel observations. Comparison between anabolic ^13^C-assimilation in the light versus the light + DCMU and night incubations, permits quantification of the symbiont boost to host anabolism via translocation. In our experiments, translocation increased ^13^C-incorporation by 13–22% in the epidermis (Fig. 2a), 40–58% in the gastrodermis (Fig. 2b), and 135–169% in gastrodermal host lipid bodies, relative to the light + DCMU and night incubations (Fig. 2c). ^13^C-labelling in the host lipid bodies in the light + DCMU and night incubations (i.e., when the recycling of metabolic ^13^CO_2_ is either blocked or inactive) has to derive from host de novo lipid synthesis. Comparison of the ^13^C-labelling levels in the light + DCMU and night treatments with those in the light, thus allows us to estimate how much host de novo lipid synthesis contributes to the total holobiont lipid reserve, when the symbiosis is functioning. We found this to be ~40% under the conditions used in this study. Finally, comparison of ^13^C-assimilation in the light + DCMU and night incubations permits to determine whether host anabolism itself exhibits diel rhythmicity; we found no statistically significant differences in the tissue regions studied (Fig. 2a–c) and thus no evidence for diel rhythmicity in anabolic turnover.

### Comparison of anabolism among structures in the coral polyp

**Fig. 3:**
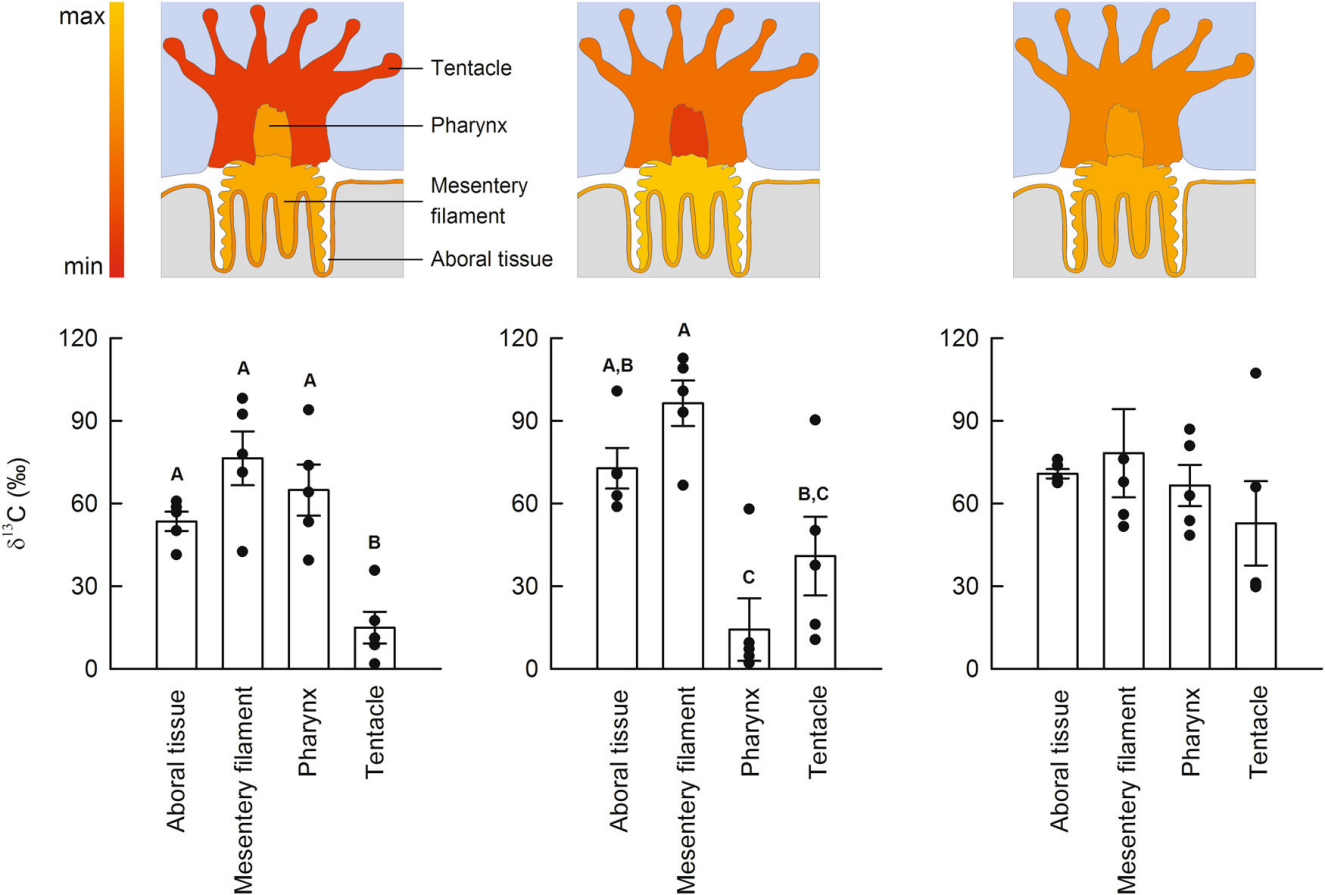
Intra-specific variation in anabolism between structures with different biological functions in a coral polyp. Individual polyps (*n* = 3) originating from corals incubated with [2,3-^13^C]-pyruvate in the light + DCMU treatment, were imaged to determine whether host anabolism alone (i.e., without the symbiont contribution) differs between regions of the polyp. Four regions of interest were identified: aboral tissue layers, mesentery filaments, pharynx and tentacle. The relative anabolic assimilation of ^13^C (denoted by majuscule letters) differed substantially between regions of interest within individual polyps. Shown are schematic representations of the allocation of ^13^C (expressed relative to the highest enrichment level measured) and the quantitative data (mean ± SE, *n* = 5 images per structure per colony).

**Fig. 4:**
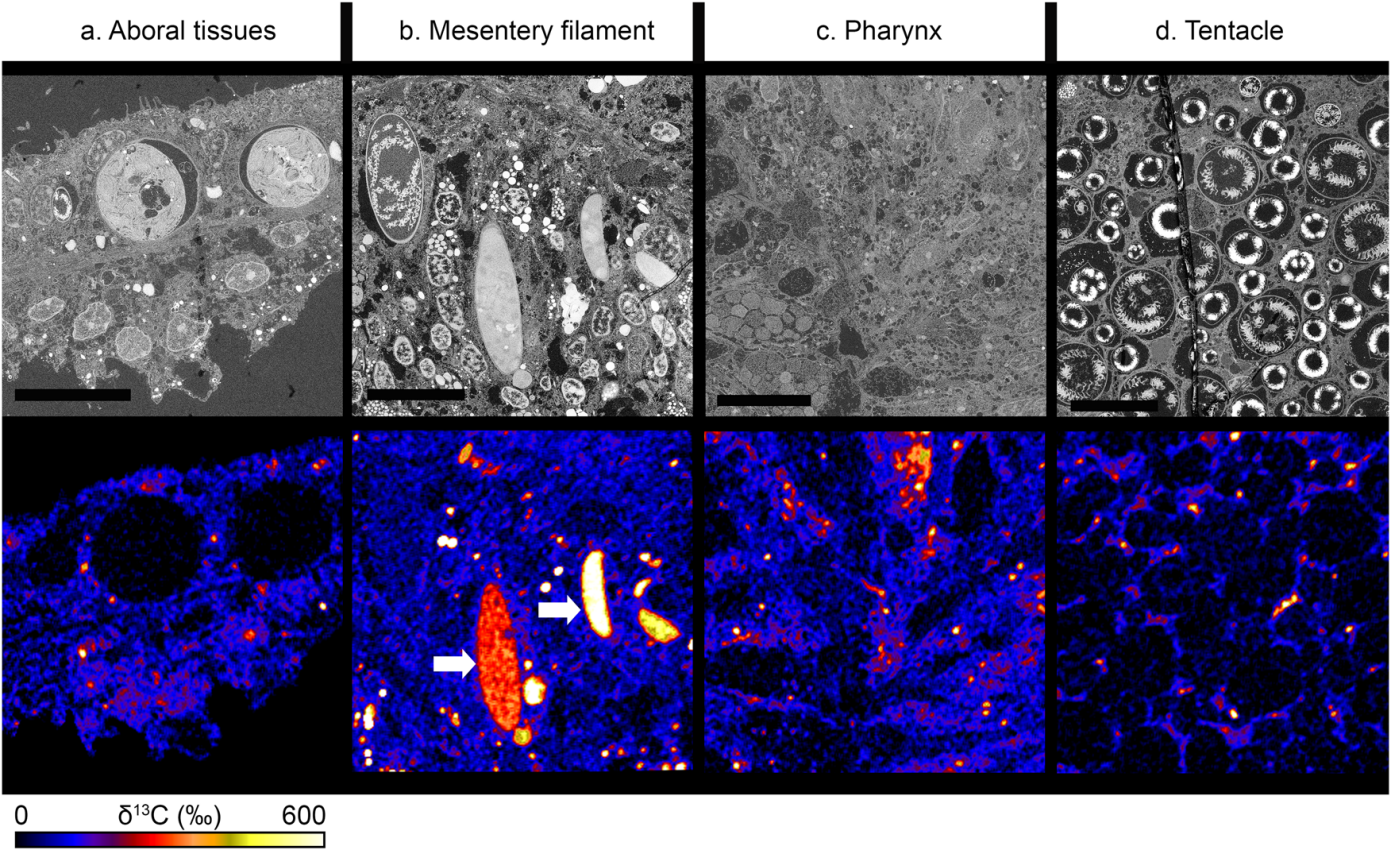
Cellular-level resolution in anabolism within structures of the coral polyp. Four regions of interest were identified in the coral polyp: the aboral tissue layers **a**, mesentery filaments **b**, pharynx **c**, and tentacle **d**. These regions were first imaged by scanning electron microscopy and then by NanoSIMS imaging. The heterogeneity of the labelling shows that there is differential anabolic activity at the level of tissue and cells. Note the localized accumulation of [2,3-^13^C]-pyruvate-derived ^13^C in secretory-type cells in the mesentery filaments (white arrows, only present in panel **b**). Scale bar represents 10 µm.

To test for differences in ^13^C-labelled pyruvate assimilation between different regions of interest (ROIs) in the coral polyp, data were first analysed by two-way ANOVA with region of interest and colony as factors (Supplementary Table 1). A significant interaction between these two-factors was detected, meaning that anabolic turnover patterns differ between polyps from different colonies (Fig. 3). Therefore individual polyps were analysed separately (Supplementary Table 2). The mesentery filaments (MFs) always had the highest levels of anabolic turnover, while the tentacles or pharynx had the lowest (Fig. 3), which is reasonable considering the localization, function and cellular composition of these structures. MFs are located in the corals gastric cavity, where they play an important role in cleaning the polyp^18^ and in the digestion of food and pathogens^19^. They contain numerous cell types including (digestive) phagocytes, (secretory) gland cell morphotypes, mucocytes, and epithelio-muscular cells^20,21^. Correlated electron microscopy (EM) revealed ^13^C was preferentially incorporated in large gland cells (Fig. 4b, white arrows), which contained heterogeneously labelled matter.

The tentacles also contain mucocytes and epithelio-muscular cells to facilitate their retraction^22^. However, as in the case of the pharynx, they are dominated by columnar support epithelial cells and nematocytes; stinging cells involved in prey capture^21^. ^13^C-enrichment was not observed in mucocytes, nor in nematocytes (Figs. 1c and  4d), suggesting that once formed, these structures require little anabolic maintenance. It is worth noting that both the acclimation period and the isotopic-labelling experiments were conducted in filtered seawater to eliminate prey from the water and prevent the confounding effects of heterotrophy on host metabolism^15,23^. It is possible that this led to reduced anabolic activity in these areas, and that regeneration or repair would have been higher if an element of feeding had been added to the experimental design.

Another interesting observation was the presence of ^13^C-rich hotspots in all of the ROIs studied (Fig. 4). Some of the hotspots (particularly those present in the MFs; Fig. 4b) were contained in vesicles, however most hotspots appeared to be free in the host tissue and outside the cell nuclei (Figs. 1 and  4); their molecular identity remains to be determined.

### Conclusions and future directions

In this study, we show that pyruvate is an effective isotopic marker of anabolism in photosymbiotic holobionts. When combined with NanoSIMS isotopic imaging, isotopically labelled pyruvate provides quantitative information at the tissue- and single cell level and can be applied to disentangle complex host–symbiont metabolic interactions. Of particular current interest, the method can be used to throw light on how reef-building corals respond to temperature-induced bleaching: i.e., when corals expel or lose their symbiotic algae^24^. An advantage of this tool is that it is broadly compatible with bulk tissue analyses, such as high-performance liquid chromatography^25^ or gas chromatography–mass spectrometry^8^, which lack spatial resolution but provide additional important information about the molecules being formed, or the specific anabolic pathways that are active.

## Methods

### Coral collection and maintenance

Three *Stylophora pistillata* mother colonies were collected in August 2018 at 8 m depth from a coral nursery situated adjacent to the Inter-University Institute for Marine Sciences (Eilat, Israel). The corals were fragmented, mounted on numbered plugs and placed in separate tanks in the Red Sea Simulator (RSS) aquarium system^26^, where they were left for a month to recover from any handling stress incurred and to acclimate to ambient conditions (Supplementary Fig. 2a). Corals were not fed during acclimation to eliminate the potentially confounding effect of heterotrophy on host metabolism^15,23^.

### Isotopic-labelling experiments

12 h isotopic pulses were conducted in 250 mL glass beakers, set atop a submersible magnetic stir-plate, which was placed in a flow-through aquarium. Day (light) and night incubations were conducted in ambient thermal conditions (26 ± 1 °C) in accordance with the diel light cycle in Eilat (day: 06:30–18:30). Light incubations were conducted under natural, but shaded light (mean: 144 ± 230 μmol photons m^−2^ s^−1^) conditions (Supplementary Fig. 2b) and used [1-^13^C]-pyruvate or [2,3-^13^C]-pyruvate (Cambridge Isotope Laboratories Tewksbury, MA, USA), with and without the photosynthetic inhibitor 3-(3,4-dichlorophenyl)-1,1-dimethylurea (DCMU). Night incubations used [1-^13^C]-pyruvate and [2,3-^13^C]-pyruvate only. Pyruvate (500 mmol stock prepared in distilled water) was added at a concentration of 1 mM. This concentration was deemed sufficient, because it produced detectable levels of labelling in NanoSIMS images in preliminary trials. DCMU (stock dissolved at 0.01% in ethanol) was added at 10 µM; a common concentration used to block photosynthesis in corals^27^. A separate experiment using fragments from the same mother colonies as those used in the isotopic-labelling experiments was conducted to ensure this concentration did not affect the respiration (and thus metabolic functioning) of the coral host (Supplementary Fig. 3).

During the isotopic-labelling experiments, water changes were performed and fresh isotopic labels were added every 3 h to ensure stable water chemistry. At the end of the labelling experiment, the apical tip of each coral fragment was removed and a 1 cm coral piece was clipped off and immersed in fixative (0.5% formaldehyde and 2.5% glutaraldehyde in 0.1 M phosphate buffer with 0.6 M sucrose, pH 7.4–7.6) for 24 h at room temperature^10^. Pieces were washed and transferred to 0.1 M phosphate buffer containing 0.5 M ethylenediaminetetraacetic acid (EDTA), where they were stored at 4 °C, until their calcium carbonate skeletons were fully dissolved (1–2 weeks).

### Sample preparation

Samples were dissected into small tissue pieces containing a single polyp and post-fixed for 1 h at room temperature with 1% osmium tetroxide in 0.1 M phosphate buffer. The samples were then dehydrated in a graded series of ethanol (50%, 70%, 90%, and 100%), and embedded in Spurr resin blocks. Thin (200 nm) and semi-thin (500 nm) sections were cut using a 45° Diatome diamond knife and mounted on round glass slides (10 mm) for scanning transmission electron microscopy (GeminiSEM 500, Carl Zeiss Microscopy GmbH, Jena, DE), or NanoSIMS imaging.

### NanoSIMS imaging

All NanoSIMS images (40 × 40 μm, 256 × 256 pixels, 5 ms pixel^−1^ dwell time, five layers) were obtained using a 16 keV Cs^+^ primary ion beam, focused to a spot-size of about 120 nm. Secondary ions (^12^C_2_^−^, ^13^C^12^C^−^) were simultaneously counted in individual electron-multiplier detectors, with a mass resolving power of ~9000 (Cameca definition). Isotopic ratios were formed from drift-corrected ion images using the ratio of ^13^C^12^C^−^ to ^12^C_2_^−^ and expressed as parts-per-thousand (‰) deviation relative to an isotopically unlabelled coral tissue sample prepared and analysed in an identical manner.

Two separate experiments were performed: (1) A *proof-of-concept* experiment, designed to quantify the different labelling patterns produced by [1-^13^C]-pyruvate and [2,3-^13^C]-pyruvate, across all experimental incubations (i.e., light, light + DCMU and night). (2) Examination of anabolic variation in different tissue regions of the coral polyp. These two experiments are presented and discussed separately below.

1. *Proof-of-concept experiment*: Analysis was limited to the oral tissue layers of the coenosarc tissue (i.e., epidermis and gastrodermis). In these two layers, it was possible to define four ROIs using the L’IMAGE software (Dr. Larry Nittler, Carnegie Institution of Washington). These ROIs were: host epidermis, host gastrodermis (excluding lipid bodies and dinoflagellates), host lipid bodies, and dinoflagellate cells. ROIs were drawn using the ^12^C^14^N^−^ images. The epidermis and gastrodermis are separated by the mesoglea and are thus easy to separate in these images. Dinoflagellate cells are also easily identified by their size and internal structure^28^. Lipid bodies are distinguished by their size and drop-like appearance and their low ^12^C^14^N^−^ signal (and hence relatively low N:C ratio, e.g. Fig. 1); these identifications have been tested numerous times against TEM images. A minimum of five images were taken of each experimental treatment (30 symbiont cells), producing a dataset that contained 138 images (Supplementary Table 3).

To test for differences in ^13^C-labelled pyruvate assimilation between [1-^13^C] and [2,3-^13^C]-pyruvate, separate Student’s *t*-tests (Bonferroni-correction: *α* = 0.004) were performed for each experimental treatment (light, light + DCMU, night) and each region of interest (epidermis, gastrodermis, host lipid bodies, symbiont; Supplementary Table 4). To test whether treatment influenced ^13^C-labelled pyruvate assimilation within a region of interest, a restricted maximum-likelihood model was used, with treatment (light, light + DCMU, night) as a fixed factor and colony as a random factor (Supplementary Table 3). If treatment was found to be significant, Tukey HSD tests were performed to identify where the differences lay. Where necessary, data were square root transformed (Supplementary Table 5) prior to analysis in order to meet the assumptions of normality (Shapiro–Wilk W test) and homogeneity of variance (Levene’s test).

2*. Examination of anabolic variation in the coral polyp*: Tissue layers in four regions of the polyp were identified as areas of interest. These were the aboral tissues (comprised of the aboral gastrodermis and the calicodermis), the tentacle, the pharynx, and the mesenteries filaments. These structures were selected because they contain highly specialized cell types adapted to their biological function. Five NanoSIMS images (same settings as above) were taken per structure for each polyp, generating a dataset of 60 images (Supplementary Table 6).

To test for patterns in pyruvate-derived ^13^C-assimilation between structures from different colonies, data were first analyzed by two-way ANOVA with structure (aboral tissues, tentacle, pharynx, mesentery filament) and colony (1–3) as fixed factors (Supplementary Table 1). Data were then split by colony and re-analyzed by one-way ANOVA and Tukey HSD tests to identify where differences lay (Supplementary Table 2). In all cases, assumptions of normality and variance equality were met.

### Reporting summary

Further information on research design is available in the Nature Research Reporting Summary linked to this article.

## Supplementary information

Supplementary Information

Reporting Summary

## Data Availability

All data generated or analysed during this study are included in this published article (and its Supplementary Information files).
