# Supplementary material for: A method to disentangle and quantify host anabolic turnover in photosymbiotic holobionts with subcellular resolution
Source: Commun Biol. 2020 Jan 8;3:14. doi: 10.1038/s42003-019-0742-6 (PMC6949218; doi:10.1038/s42003-019-0742-6)
Supplement: Supplementary file 1 — Supplementary Information [file 42003_2019_742_MOESM1_ESM.pdf]

Supplementary Figures

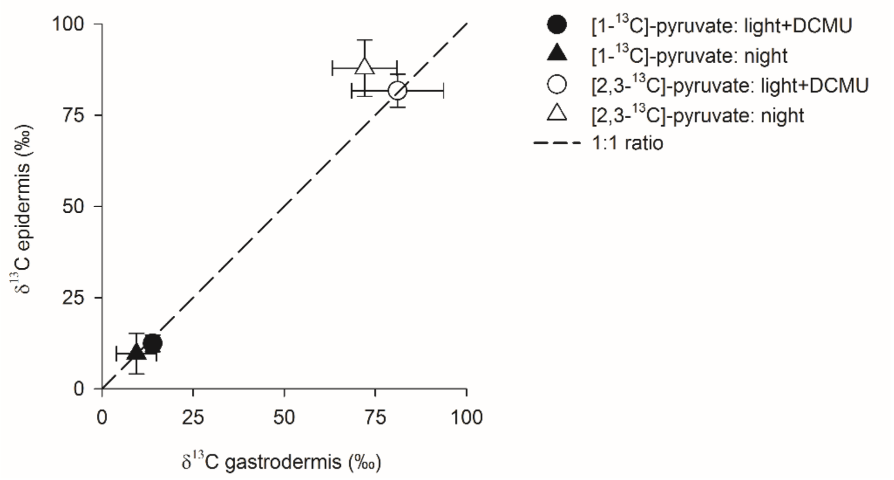

Supplementary Fig. 1. ^{13}C-assimilation in the epidermis relative to the gastrodermis in the light+DCMU and night incubations. The congruence between the two suggests the two tissue layers have access to, and assimilate pyruvate equally. Dashed line denotes 1:1 ratio.

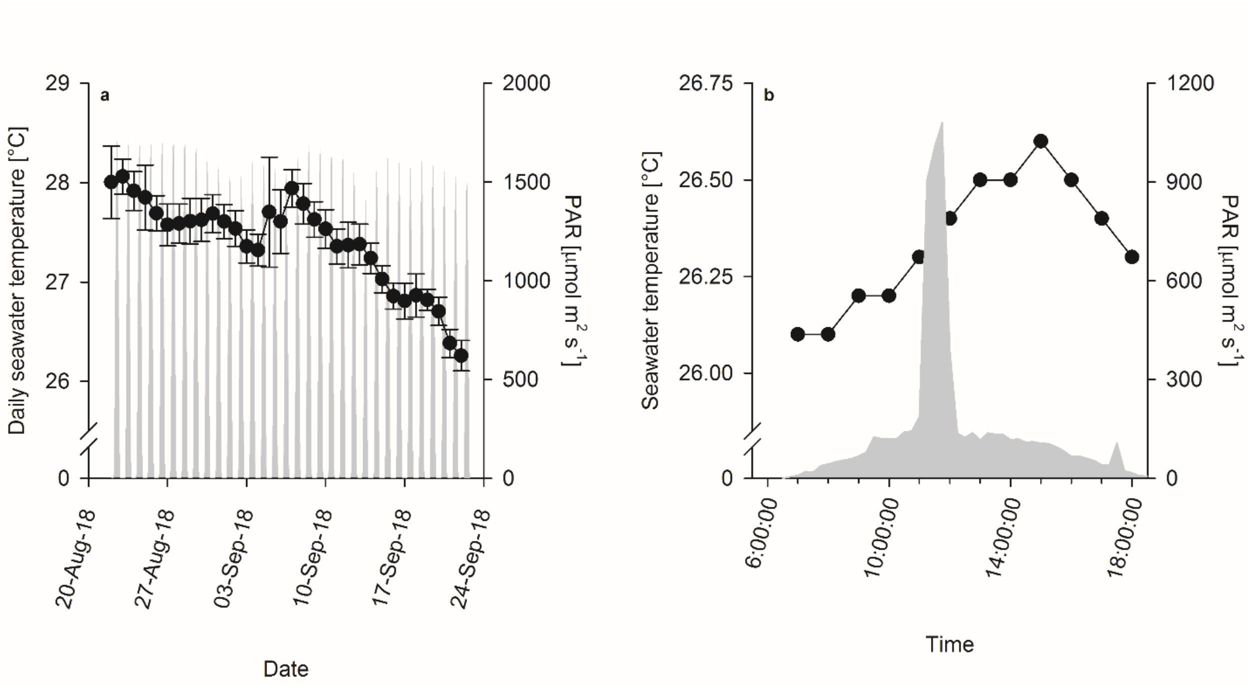

Supplementary Fig. 2. Environmental conditions. Three coral colonies were collected on the 22nd of August, 2018. The corals were fragmented (4 cm pieces) mounted on numbered plugs and placed in separate tanks in the Red Sea Simulator for one month before the experiments were conducted. Shown: (a) daily temperature (mean ± s.d) and light conditions experienced during the month-long acclimation phase (data collected by The Israel National Monitoring Program in the Gulf of Eilat: http://www.iui-eilat.ac.il/Research/NMPMeteoData.aspx); and (b) hourly temperature (mean) and light conditions during the light and light+DCMU isotopic pulses.

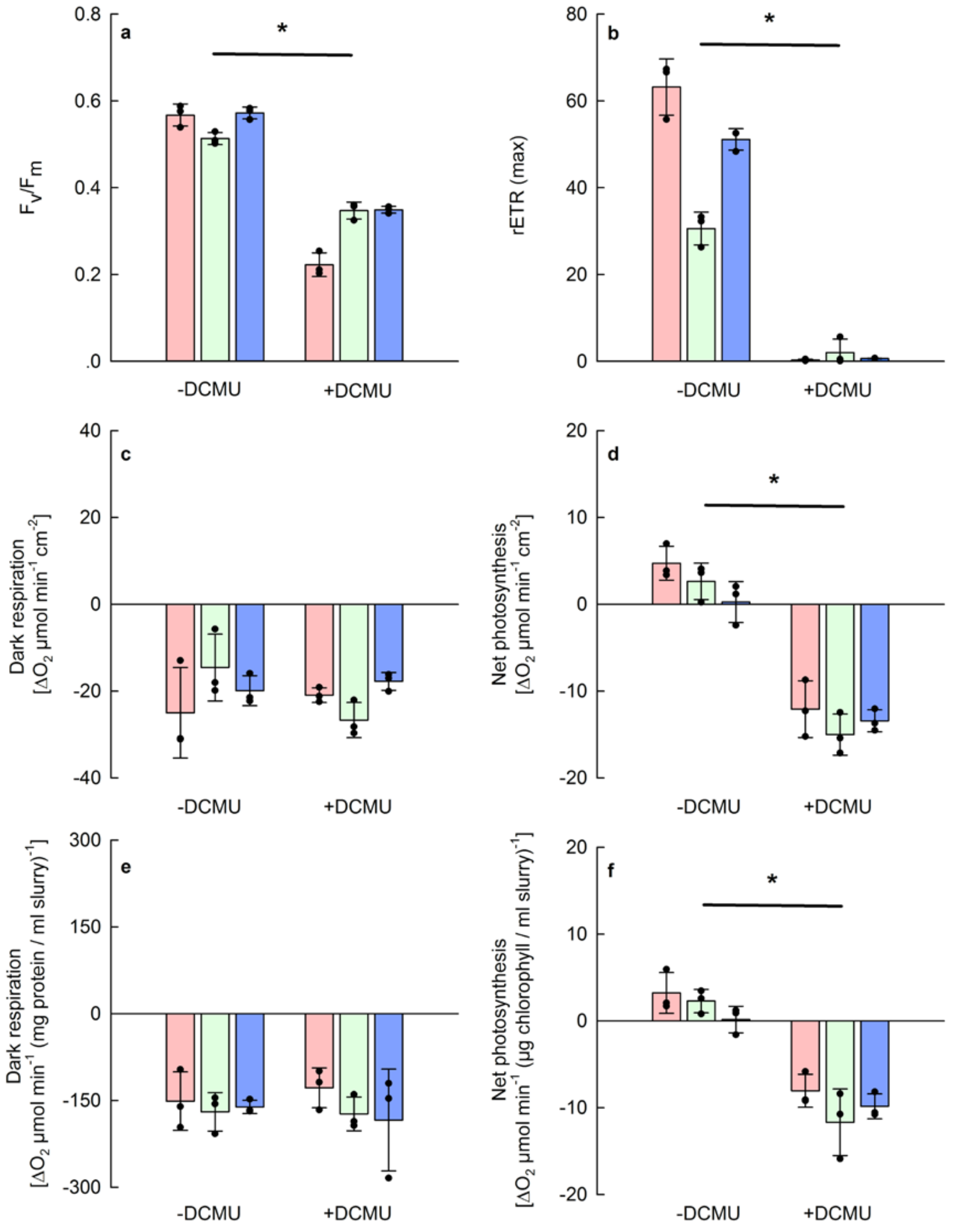

Supplementary Fig. 3. Assessing the effects of 10 µm DCMU on coral physiology. a. maximum quantum yield of photosystem II (F_v/F_m); b. maximum relative electron transport rate (rETR max); c. dark respiration normalized to the surface area of the coral. d. net photosynthesis normalized to the surface area of the coral. e. respiration normalized by host protein content. f. net photosynthesis normalized to total chlorophyll content. Note that measurements were conducted in triplicate (i.e. $n = 3$ fragments *per* colony), and bars show mean ± s.d. The three biological replicates (i.e. colonies) are coloured differently. Asterisks represent significant differences (Students t-test; $p < 0.05$) between treatments. The methodology used to derive these results and an expanded interpretation of the results is provided in the Supplementary Methods and Discussion section.

Supplementary Tables

Supplementary Table 1. Statistical output showing differences in ^{13}C-assimilation between structures in the coral polyp. Shown is the outcome of a two-way ANOVA conducted to test whether there is a common pattern in anabolism between regions of interest in polyps from different coral colonies. Statistically-significant factors ($p < 0.005$) are highlighted in bold.

**Source**	**DF**	**F-ratio**	***p*-value**
Colony	2	2.2762	0.1137
Region of interest	3	12.5284	**< 0.0001**
Colony × Region of interest	6	4.1028	**0.0021**

Supplementary Table 2. Statistical output showing differences in ^{13}C-assimilation between structures in individual polyps. Shown is the outcome of a three one-way ANOVAs conducted to test whether anabolism varies in different structural components of the coral polyp. Statistically-significant factors ($p < 0.005$) are highlighted in bold.

**Source**	**Colony**	**DF**	**F-ratio**	***p*-value**
Region of interest	1	3	12.6075	**0.0002**
	2	3	11.4329	**0.0003**
	3	3	0.8345	0.4944

Supplementary Table 3. Summary of ^{13}C-enrichment values from the *proof-of concept* experiment. Regions of interest (ROI) were based on coenosarc cross sections from the oral tissue layer. Gastrodermis ROIs refer to the gastrodermis tissue with symbionts and lipids subtracted.

Treatment	Isotope	Colony	ROI	δ^{13}C mean	δ^{13}C SD	N
Light	[1-^{13}C]-pyruvate	1	Epidermis	14.442	1.224	8
			Gastrodermis	35.700	3.400	8
			Host lipid bodies	114.451	38.172	26
			Symbiont	178.146	56.550	31
		2	Epidermis	8.788	5.281	5
			Gastrodermis	20.755	5.065	5
			Host lipid bodies	143.215	55.493	13
			Symbiont	152.759	65.879	35
		3	Epidermis	11.672	8.142	7
			Gastrodermis	31.585	13.542	7
			Host lipid bodies	178.045	124.155	13
			Symbiont	135.489	56.102	36
	[2,3-^{13}C]-pyruvate	1	Epidermis	96.118	15.625	8
			Gastrodermis	98.884	11.501	8
			Host lipid bodies	155.134	58.930	16
			Symbiont	152.671	58.957	34
		2	Epidermis	91.623	17.114	8
			Gastrodermis	99.059	12.525	8
			Host lipid bodies	132.185	40.214	8
			Symbiont	105.152	43.689	35
		3	Epidermis	110.928	26.576	9
			Gastrodermis	142.865	6.991	9
			Host lipid bodies	162.149	67.148	58
			Symbiont	329.342	100.656	33
Light+DCMU	[1-^{13}C]-pyruvate	1	Epidermis	8.402	4.422	7
			Gastrodermis	13.439	4.816	7
			Host lipid bodies	10.105	11.115	12
			Symbiont	6.086	6.103	30
		2	Epidermis	12.694	4.538	7
			Gastrodermis	11.007	6.430	7
			Host lipid bodies	11.750	16.785	6
			Symbiont	7.044	8.020	33
		3	Epidermis	16.294	4.446	11
			Gastrodermis	17.189	3.825	11
			Host lipid bodies	10.500	15.385	25
			Symbiont	9.205	7.474	29
	[2,3-^{13}C]-pyruvate	1	Epidermis	73.549	9.910	7
			Gastrodermis	67.741	18.487	7
			Host lipid bodies	69.132	21.985	6
			Symbiont	7.169	13.679	36
		2	Epidermis	89.109	9.208	6
			Gastrodermis	69.224	3.563	7
			Host lipid bodies	71.058	40.669	11
			Symbiont	15.246	12.396	34
		3	Epidermis	82.469	16.044	9
			Gastrodermis	106.231	20.965	9
			Host lipid bodies	50.894	30.517	21
			Symbiont	3.414	12.048	33

Night	[1-^{13}C]-pyruvate	1	Epidermis	-1.534	3.384	8
			Gastrodermis	-1.472	2.918	8
			Host lipid bodies	-1.114	9.704	2
			Symbiont	-6.692	8.737	31
		2	Epidermis	15.014	1.974	8
			Gastrodermis	13.991	1.842	8
			Host lipid bodies	12.845	10.669	16
			Symbiont	9.403	6.158	34
		3	Epidermis	15.391	5.107	8
			Gastrodermis	15.713	4.137	8
			Host lipid bodies	12.423	15.843	25
			Symbiont	10.706	8.334	33
	[2,3-^{13}C]-pyruvate	1	Epidermis	87.402	9.988	8
			Gastrodermis	62.680	12.610	8
			Host lipid bodies	63.386	12.813	16
			Symbiont	12.621	6.333	40
		2	Epidermis	101.525	10.142	6
			Gastrodermis	63.643	13.617	6
			Host lipid bodies	52.212	16.250	6
			Symbiont	6.641	6.585	40
		3	Epidermis	74.691	17.058	8
			Gastrodermis	89.726	11.330	8
			Host lipid bodies	51.425	30.359	38
			Symbiont	9.325	10.217	36
Unlabelled	None	n/a	Epidermis	-0.323	1.583	15
			Gastrodermis	-0.101	1.113	15
			Host lipid bodies	0.687	13.843	19
			Symbiont	1.029	7.766	82

Supplementary Table 4. Statistical output of the differences in ^{13}C-assimilation between [1-^{13}C] and [2,3-^{13}C]-pyruvate. Shown is the outcome of multiple student's t-tests conducted for each experimental treatment and each region of interest (oral tissue layers of the coral coenosarc). Bonferroni-corrected ($\alpha < 0.004$) statistically-significant differences are highlighted in bold here, and with asterisks on Figure 2.

Region of interest	Treatment	Test outcome
Epidermis	Light	t(28) = 19.5490, ***p* = <0.0001**
	Light+DCMU	t(27) = 22.1164, ***p* = <0.0001**
	Night	t(31) = 19.4811, ***p* = <0.0001**
Gastrodermis	Light	t(34) = 15.9961, ***p* = <0.0001**
	Light+DCMU	t(24) = 13.0641, ***p* = <0.0001**
	Night	t(21) = 9.8528, ***p* = <0.0001**
Host lipid bodies	Light	t(101) = 1.1746, *p* = 0.8785
	Light+DCMU	t(64) = 8.3225, ***p* = <0.0001**
	Night	t(95) = 10.7870, ***p* = <0.0001**
Symbiont	Light	t(170) = 2.0843, *p* = 0.9807
	Light+DCMU	t(160) = 0.7933, *p* = 0.7856
	Night	t(150) = 0.2051, *p* = 0.5811

Supplementary Table 5. Statistical output showing differences in ^{13}C-assimilation between treatments. Shown are the output from a restricted maximum-likelihood model, using treatment as a fixed factor and colony as a random factor (variance components analysis). Any transformations to the data (required to meet the conditions of normality and homogeneity of variance) are described. Statistically-significant differences ($p < 0.05$) are shown in bold here, and the results of the least squares means Tukey HSD *post-hoc* tests are indicated using letters on Figure 2.

Isotope	Transformation	Region of interest	Treatment	Colony
[1-^{13}C]-pyruvate	-	Epidermis	$F_{2,64}$ = 1.7544, *p* = 0.1812	27%
	-	Gastrodermis	$F_{2,64}$ = 43.4161, ***p* < 0.0001**	12%
	Square root	Host lipid bodies	$F_{2,132}$ = 107.8197, ***p* < 0.0001**	7%
	Square root	Symbiont	$F_{2,244}$ = 808.1129, ***p* < 0.0001**	2%
[2,3-^{13}C]-pyruvate	Square root	Epidermis	$F_{2,64}$ = 6.7017, ***p* = 0.0023**	0%
	-	Gastrodermis	$F_{2,65}$ = 64.0241, ***p* < 0.0001**	71%
	-	Host lipid bodies	$F_{2,183}$ = 98.8799, ***p* < 0.0001**	0%
	Square root	Symbiont	$F_{2,316}$ = 312.9260, ***p* < 0.0001**	26%

Supplementary Table 6. Summary of ^{13}C-enrichment values between different structures in the coral polyp. Regions of interest (ROI) were taken in polyps from the light+DCMU treatment. This allows for comparison of host anabolism (without the contribution of the algal symbiont), between structures with different biological functions.

ROI	Colony	$\delta^{13}C$		N
		**Mean**	**S.D**	
Aboral tissues	1	53.577	7.881	5
	2	72.857	16.436	5
	3	70.837	3.770	5
Mesentery filament	1	76.442	21.793	5
	2	96.443	18.350	5
	3	78.325	35.879	5
Pharynx	1	64.920	20.674	5
	2	14.273	25.341	5
	3	66.564	16.789	5
Tentacle	1	14.964	12.879	5
	2	40.966	31.898	5
	3	52.835	34.097	5

Supplementary Methods and Discussion

Experimental overview

Three coral fragments from each colony were selected at random and transferred to plastic containers containing 0.22 µm filtered seawater only (i.e. -DCMU; n = 3 *per* colony, n = 3 colonies). A further three fragments from each colony were transferred to plastic containers containing 0.22 µm filtered seawater and 10 µM of DCMU (+DCMU, n = 3 *per* colony, n = 3 colonies). Corals were maintained in these respective treatments (i.e. ± DCMU) while live measurements were obtained. The effects of DCMU on coral photo-physiology (F_v/F_m and rETR max) were first assessed using PAM fluorometry. Dark respiration and net photosynthesis were then measured in the same fragments using the O_2 evolution method. At the end of the incubations, all fragments were snap-frozen in liquid nitrogen and stored at -80 °C for determination of surface area, host protein content and chlorophyll content. See sections below for details of the protocols used.

PAM fluorometry

Coral fragments were dark-adapted for a minimum of 15 mins; a time-period that is sufficient to stabilize dark-adapted F_v/F_m values[1]. Rapid light curves[2] (RLC) were generated using an imaging pulse amplitude modulated fluorometer (Heinz Walz GmbH, Effeltrich, Germany), with measurements taken at 30 s intervals ranging from 0 to 701 µmol m^{-2} s^{-1}. Settings (MI: 2, SI: 10, Gain: 2, Damping: 2) were kept consistent between replicate experimental runs to ensure the results between treatments were comparable. The maximum quantum yield of PSII (F_v/F_m; Fig. S2a) was derived from the first yield measurement of the RLC, and maximum relative electron transport rate (rETR max; Fig. S2b) was identified as the maximum value obtained after multiplying the maximum yield of PSII (YII) by the photosynthetically active radiation (PAR).

Dark respiration and net photosynthesis

Dark respiration and net photosynthesis were measured in sealed, stir bar-agitated, glass jars equipped with oxygen optodes that were connected to one of two OXY-4 mini oxygen meters (Precision Sensing GmbH, Regensburg, Germany), capable of recording the oxygen saturation state of the water (%) every second. Dark respiration rates were calculated from the linear slope of the change in relative oxygen saturation *per* minute (ΔO_2 min^{-1}), which was obtained during a period of time ranging from 20 minutes up to an hour (i.e., until a constant slope was observed). 20 min dark-adaptation period is normally sufficient to fully oxidize the plastoquinone (PQ) pool and thus, avoid any light enhanced respiration rates[3]. Net photosynthesis was calculated from the linear slope of the change in relative oxygen saturation *per* minute (ΔO_2 min^{-1}) after 15 mins adaption to 100 µmol photons m^{-2} s^{-1} of white light. Dark respiration rates were subsequently normalized to both coral surface area (Fig. S2c) and host protein content (Fig. S2e), while net photosynthesis values were normalized to surface area (Figs. S2d) and total chlorophyll content (Fig. S2f).

Calculation of normalization factors; host protein content, total chlorophyll and surface area

Frozen samples were thawed and the coral tissue was brushed into ice-cold phosphate buffered saline (PBS) using a waterpik. The resulting tissue slurry was homogenized (30 s; DIAX 100, Heidolph Instruments, Schwabach, Germany) and centrifuged (10 min, 5000 × g, 4 °C) in order to separate the host tissue and the algal symbionts.

The host-tissue containing supernatant was decanted, vortexed, and an aliquot was removed for analysis of host protein content using the improved Bradford assay with bovine serum albumin (BSA) as the standard[4]. The algal pellet was washed twice in PBS (10 min, 5000 × *g*, 4 °C), and then re-suspended in 1 mL of 100 % acetone for chlorophyll extraction (24 h at 4 °C). Total chlorophyll content was determined from the fluorescence measured at 630, 663, and 750 nm, and applied to dinoflagellate-specific equations[5]. The remaining skeletons of the coral fragments were left to dry (48 h) and then measured for the surface area *via* the paraffin wax dipping method[6].

Results and interpretation:

DCMU addition had a strong and significant effect on all physiological parameters tested, except for dark respiration, which remained stable with or without DCMU (Fig. S2). The flux of electrons from PSII to PSI (i.e. rETR max) was completely blocked by +DCMU (Fig. 2b). DCMU is a known inhibitor of photosynthesis, which blocks the PQ binding site of PSII, preventing the transfer of electrons and protons to the PQ pool. In this state, electrons are no longer transferred to the rest of the photosynthetic chain (plastocyanine and cytochrome b6f), causing an overall inactivation of photosynthesis. Our rETR max results (Fig. S2b) validate the efficiency of DCMU in blocking the electron transport. As a result, net photosynthesis (Figs. 2d, f) was also cancelled in the +DCMU treatment, compared to the -DCMU. A negative rate of oxygen relates to a consumption of oxygen rather than a production. We therefore conclude that +DCMU corals no longer perform photosynthesis. Dark respiration (Figs. 2 c, e) was not affected by +DMCU, with values similar to the negative controls for both normalization parameters (i.e. surface area; Fig. 2c, and host protein content; Fig. 2e). Corals exposed to DCMU displayed levels F_v/F_m twice as low as for the corals without DCMU (Fig. 2a). The mechanism of chlorophyll fluorescence is independent of the action that DCMU has on PQ, which takes place in PSII. It is not possible from our results to explain why dark respiration is higher in magnitude than net photosynthesis in the +DCMU treatment, but this would be an interesting subject for future studies to investigate.

References

1 Nakamura, T., Van Woesik, R. & Yamasaki, H. Photoinhibition of photosynthesis is reduced by water flow in the reef-building coral Acropora digitifera. *Mar. Ecol. Prog. Ser.* **301**, 109-118 (2005).

2 Ralph, P. J. & Gademann, R. Rapid light curves: a powerful tool to assess photosynthetic activity. *Aquat. Bot.* **82**, 222-237 (2005).

3 Moberg, F., Nyström, M., Kautsky, N., Tedengren, M. & Jarayabhand, P. Effects of reduced salinity on the rates of photosynthesis and respiration in the hermatypic corals *Porites lutea* and *Pocillopora damicornis*. *Mar. Ecol. Prog. Ser.* **157**, 53-59 (1997).

4 Zor, T. & Selinger, Z. Linearization of the Bradford protein assay increases its sensitivity: theoretical and experimental studies. *Anal. Biochem*. **236**, 302-308 (1996).

5 Jeffrey, S. & Humphrey, G. New spectrophotometric equations for determining chlorophylls *a*, *b*, c1 and c2 in higher plants, algae and natural phytoplankton. *Biochem. Physiol. Pflanz.* **167**, 191-194 (1975).

6 Stimson, J. & Kinzie III, R. A. The temporal pattern and rate of release of zooxanthellae from the reef coral *Pocillopora damicornis* (Linnaeus) under nitrogen-enrichment and control conditions. *J. Exp. Mar. Biol. Ecol.* **153**, 63-74 (1991).
