# Supplementary material for: A method to disentangle and quantify host anabolic turnover in photosymbiotic holobionts with subcellular resolution
Source: Commun Biol. 2020 Jan 8;3:14. doi: 10.1038/s42003-019-0742-6 (PMC6949218; doi:10.1038/s42003-019-0742-6)
Supplement: Supplementary file 2 — Reporting Summary [file 42003_2019_742_MOESM2_ESM.pdf]

natureresearch

Corresponding author(s): Anders Meibom

Last updated by author(s): Dec 3, 2019

Reporting Summary

Nature Research wishes to improve the reproducibility of the work that we publish. This form provides structure for consistency and transparency in reporting. For further information on Nature Research policies, see Authors & Referees and the Editorial Policy Checklist.

Statistics

For all statistical analyses, confirm that the following items are present in the figure legend, table legend, main text, or Methods section.

n/a	Confirmed	
☐	☒	The exact sample size (*n*) for each experimental group/condition, given as a discrete number and unit of measurement
☐	☒	A statement on whether measurements were taken from distinct samples or whether the same sample was measured repeatedly
☒	☐	The statistical test(s) used AND whether they are one- or two-sided<br>*Only common tests should be described solely by name; describe more complex techniques in the Methods section.*
☐	☒	A description of all covariates tested
☐	☒	A description of any assumptions or corrections, such as tests of normality and adjustment for multiple comparisons
☐	☒	A full description of the statistical parameters including central tendency (e.g. means) or other basic estimates (e.g. regression coefficient) AND variation (e.g. standard deviation) or associated estimates of uncertainty (e.g. confidence intervals)
☐	☒	For null hypothesis testing, the test statistic (e.g. *F*, *t*, *r*) with confidence intervals, effect sizes, degrees of freedom and *P* value noted<br>*Give P values as exact values whenever suitable.*
☒	☐	For Bayesian analysis, information on the choice of priors and Markov chain Monte Carlo settings
☒	☐	For hierarchical and complex designs, identification of the appropriate level for tests and full reporting of outcomes
☒	☐	Estimates of effect sizes (e.g. Cohen's *d*, Pearson's *r*), indicating how they were calculated

Our web collection on statistics for biologists contains articles on many of the points above.

Software and code

Policy information about availability of computer code

Data collection	n/a
Data analysis	n/a

For manuscripts utilizing custom algorithms or software that are central to the research but not yet described in published literature, software must be made available to editors/reviewers. We strongly encourage code deposition in a community repository (e.g. GitHub). See the Nature Research guidelines for submitting code & software for further information.

Data

Policy information about availability of data

All manuscripts must include a data availability statement. This statement should provide the following information, where applicable:

- Accession codes, unique identifiers, or web links for publicly available datasets
- A list of figures that have associated raw data
- A description of any restrictions on data availability

All data generated or analysed during this study are included in this published article (and its Supplementary Information files).

Field-specific reporting

Please select the one below that is the best fit for your research. If you are not sure, read the appropriate sections before making your selection.

☐ Life sciences ☐ Behavioural & social sciences ☒ Ecological, evolutionary & environmental sciences

For a reference copy of the document with all sections, see nature.com/documents/nr-reporting-summary-flat.pdf

Ecological, evolutionary & environmental sciences study design

All studies must disclose on these points even when the disclosure is negative.

Study description	The goal of the study was to quantify coral anabolism, with and without the contribution of their symbiotic dinoflagellate partners. To do this, we used two forms of 13C-labeled pyrvuate ([1-13C]-pyruvate and [2,3-13C]-pyruvate) and three experimental treatments (light, light+DCMU and night). Three independent replicates originating from separate coral mother colonies (i.e. n = 3) were exposed to each treatment.
Research sample	Three Stylophora pistillata colonies were collected in August 2018 at 8 m depth from a coral nursery situated adjacent to the Inter-University Institute for Marine Sciences (Eilat, Israel). This location was chosen because: 1) it can be safely accessed from the shore; 2) it is not far from the laboratory and thus does not subject the corals to long (and stressful) transportation times and 3) long-term temperature and light recordings are available from this location so we know the environmental history of the samples.
Sampling strategy	Corals were fragmented, mounted on numbered plugs and placed in separate tanks in the Red Sea Simulator (RSS) aquarium system, where they were left for a month to recover from any handling stress incurred and to acclimate to ambient conditions. N=3 was deemed sufficient because labeling patterns were either present or absent and thus the results were expected to be unambiguous (e.g. with DCMU and/or at night there was zero labeling in the symbionts).
Data collection	Data was acquired using the NanoSIMS by Dr. Emma Gibbin over a period of three months. Images were taken of isotopically unlabelled coral tissue (prepared and measured in an identical manner) at the start of each day of analysis in order to provide isotopic controls and to check instrument performance. The NanoSIMS instrument was always tuned to a minimum mass resolving power of >8000 (Cameca definition; enough to avoid interferences in the mass-spectrum) and settings (dwell time, number of pixels and number of layers) were kept constant between images to ensure data gained from different treatments were comparable. images.
Timing and spatial scale	The isotopic labeling pulse experiments were conducted within a period of 24 h; light and light+DCMU were conducted during the day (i.e. from 6:00-18:00) and the night were conducted in the 12 h following that. The samples were fixed at the end of the pulse. Post-processing (dissolution of the skeleton, osmication; dehydration, embedding in epoxy etc.) was conducted simultaneously, within one month of the completion of the experiment. Once samples are in blocks they are stable and can thus be processed at a later date.
Data exclusions	No data were excluded from the study.
Reproducibility	No attempts were made to repeat the experiment because all attempts were successful first time. A high level of technical replication was implemented for the NanoSIMS, with a minimum of 30 symbionts imaged per experimental treatment, per coral replicate. This replication level was sufficient to counter-act any variation introduced during microtoming.
Randomization	Allocation was not random; three separate mother colonies were fragmented before the experiment and tagged so that one fragment from each mother colony could be exposed to each treatment (i.e. biological replication level of N=3). The fragment from each mother colony however, was selected at random using a random-number generator.
Blinding	No blinding was used during data analysis. This was not necessary since values are expressed relative to the isotopically unlabelled coral tissue measured at the start of each day of analysis, and cannot thus be misinterpreted or mishandled.

Did the study involve field work? ☒ Yes ☐ No

Field work, collection and transport

Field conditions	Seawater temperatures averaged 27.4 degrees during the acclimation period and light levels during the day (i.e. 6:00-18:00) averaged 921 µmol m-2 sec-1
Location	Corals were collected at a depth of 8m, from a coral nursery situated adjacent to the Inter-University Institute for Marine Sciences in Eilat, Israel (29°30'05.0"N 34°55'02.0"E).
Access and import/export	All corals were collected under permit 2013/40158 of the Israel Nature and Parks Authority, designated to Prof. Maoz Fine (Bar Ilan University and the Interuniversity Institute for Marine Sciences, Israel).
Disturbance	The coral nursery was set-up and maintained in an area just aside the reef so as to prevent any disturbance to the actual reef.

Reporting for specific materials, systems and methods

We require information from authors about some types of materials, experimental systems and methods used in many studies. Here, indicate whether each material, system or method listed is relevant to your study. If you are not sure if a list item applies to your research, read the appropriate section before selecting a response.

Materials & experimental systems

n/a	Involved in the study	
☒	☐	Antibodies
☒	☐	Eukaryotic cell lines
☒	☐	Palaeontology
☐	☒	Animals and other organisms
☒	☐	Human research participants
☒	☐	Clinical data

Methods

n/a	Involved in the study	
☒	☐	ChIP-seq
☒	☐	Flow cytometry
☒	☐	MRI-based neuroimaging

Animals and other organisms

Policy information about studies involving animals; ARRIVE guidelines recommended for reporting animal research

Laboratory animals	The study did not involve laboratory animals.
Wild animals	The study did not involve wild animals.
Field-collected samples	Corals were kept in separate tanks in the Red Sea Simulator (RSS) aquarium system for one month to recover from any handling stress incurred and to acclimate to ambient conditions (temperature = 27.4 degrees, light 6:00-18:00 = 921 µmol m-2 sec-1)
Ethics oversight	No ethics approval is required for marine invertebrates.

Note that full information on the approval of the study protocol must also be provided in the manuscript.
